# Association between emergency medical service transport time and survival in patients with traumatic cardiac arrest: a Nationwide retrospective observational study

**DOI:** 10.1186/s12873-021-00499-z

**Published:** 2021-09-16

**Authors:** Hiromichi Naito, Tetsuya Yumoto, Takashi Yorifuji, Tsuyoshi Nojima, Hirotsugu Yamamoto, Taihei Yamada, Kohei Tsukahara, Mototaka Inaba, Takeshi Nishimura, Takenori Uehara, Atsunori Nakao

**Affiliations:** 1 Department of Emergency, Critical Care, and Disaster Medicine, Dentistry and Pharmaceutical Sciences, Okayama University Graduate School of Medicine, 2-5-1 Shikatacho, Kitaku, Okayama, 700-8558 Japan; 2 Dentistry and Pharmaceutical Sciences, Department of Epidemiology, Okayama University Graduate School of Medicine, Okayama, Japan; 3 Hyogo Emergency Medical Center, Department of Emergency and Critical Care Medicine, Kobe, Hyogo Japan; 4 Dentistry and Pharmaceutical Sciences, Department of Orthopaedic Surgery, Okayama University Graduate School of Medicine, Okayama, Japan

**Keywords:** Mortality, Trauma care, Cardiac arrest, Time-to-treatment

## Abstract

**Background:**

Patients with traumatic cardiac arrest (TCA) are known to have poor prognoses. In 2003, the joint committee of the National Association of EMS Physicians and the American College of Surgeons Committee on Trauma proposed stopping unsuccessful cardiopulmonary resuscitation (CPR) sustained for > 15 min after TCA. However, in 2013, a specific time-limit for terminating resuscitation was dropped, due to the lack of conclusive studies or data. We aimed to define the association between emergency medical services transport time and survival to demonstrate the survival curve of TCA.

**Methods:**

A retrospective review of the Japan Trauma Data Bank. Inclusion criteria were age ≥ 16, at least one trauma with Abbreviated Injury Scale score (AIS) ≥ 3, and CPR performed in a prehospital setting. Exclusion criteria were burn injury, AIS score of 6 in any region, and missing data. Estimated survival rate and risk ratio for survival were analyzed according to transport time for all patients. Analysis was also performed separately on patients with sustained TCA at arrival.

**Results:**

Of 292,027 patients in the database, 5336 were included in the study with 4141 sustained TCA. Their median age was 53 years (interquartile range (IQR) 36–70), and 67.2% were male. Their median Injury Severity Score was 29 (IQR 22–41), and median transport time was 11 min (IQR 6–17). Overall survival after TCA was 4.5%; however, survival of patients with sustained TCA at arrival was only 1.2%. The estimated survival rate and risk ratio for sustained TCA rapidly decreased after 15 min of transport time, with estimated survival falling below 1%.

**Conclusion:**

The chances of survival for sustained TCA declined rapidly while the patient is transported with CPR support. Time should be one reasonable factor for considering termination of resuscitation in patients with sustained TCA, although clinical signs of life, and type and severity of trauma should be taken into account clinically.

## Background

Patients with traumatic cardiac arrest (TCA) have poor prognoses, and resuscitation after TCA is sometimes considered futile due to low survival rates and poor neurological outcomes [1]. Efforts to save patients with TCA can consume prehospital, emergency department (ED), intensive care unit, and surgical resources, including blood products and surgical supplies.

In 2003, the Joint Committee of the National Association of EMS Physicians (NAEMSP) and the American College of Surgeons Committee on Trauma (ACSCOT) recommended stopping cardiopulmonary resuscitation (CPR) in patients with TCA after 15 min of unsuccessful CPR [2]. The NAEMSP/ACSCOT guideline was updated in 2013 [3], when a specific time-limit for terminating resuscitation was dropped due to the lack of conclusive studies or data. Past studies indicate that TCA patients with longer prehospital time while in arrest are not likely to survive; however, the length of time for resuscitation of patients with TCA is described only on the small number of TCA survivors. Patients with critical trauma present with hypovolemia and low cardiac output, followed by a loss of their palpable pulse, making time measurement after TCA difficult. Research supporting termination for resuscitation efforts and quantitative evaluation of the impact of prehospital time on survival of patients with TCA is still needed. In this study, we examined the association between emergency medical services (EMS) transport time and survival using a large database to demonstrate the survival curve, and suggest a critical time window for successful TCA care.

## Methods

### Study design

We performed a retrospective observational study of the Japan Trauma Data Bank (JTDB), a multi-institutional database. In 2018, 280 major emergency medical institutions across Japan, similar to Level I trauma centers in the United States, were contributing to the database [4]. The JTDB data is collected through a web-based form and consists of age, sex, mechanism of injury, Abbreviated Injury Scale (AIS) using code version 1998, Injury Severity Score (ISS), probability of survival using the Trauma Injury Severity Score (TRISS) method [5], prehospital treatments including CPR, cardiac arrest (CA) status on hospital arrival, treatment dates, several time points from hospital arrival to discharge, and survival at discharge. The emergency physicians or surgeons registered the data in cooperation with medical assistants. The study was approved by the Ethics Committee of the Okayama University (K2001–004) and it conforms to the provisions of the Declaration of Helsinki. Patient consent was waived by this committee.

### Emergency medical service system

In Japan, nearly all CA cases are transported by public EMS employed by the Fire and Disaster Management Agency. All EMS personnel can perform basic life support according to the Japanese CPR guidelines, which basically conform to the American Heart Association guidelines, including use of bag-valve-mask (BVM) ventilation and automated defibrillators. An ambulance generally has a crew of three EMS personnel with at least one emergency life-saving technician (ELST). ELSTs can use supraglottic devices. Additionally, ELSTs who complete the additional training may perform endotracheal intubation. Similarly, ELSTs with additional training may establish intravenous (IV) lines and administer adrenaline. Physician-staffed ambulances run only in limited areas. EMS personnel in Japan are not allowed to perform other advanced interventions (eg, surgical airway, chest drain, or intraosseous access). Severe trauma cases are generally encouraged to be transported to trauma centers; however, TCA cases may sometimes be transported to other types of emergency medical centers to minimize transport time. EMS personnel generally transport a wide range of TCA cases to hospitals, except for definite deaths like decapitation, torso transection, or rigor mortis. Prehospital death pronouncement is not common in Japan for cases where CPR is initiated; currently, there are no protocols for termination of resuscitation in the field.

### Selection of participants

The database was queried for patients ≥16 years of age, treated from January 2004 to December 2017, who presented with major trauma (AIS score ≥ 3) in at least one body region, and CPR performed in a prehospital setting for inclusion in the study. Patients with burns, AIS score of 6 (the maximum lethal level, who would not survive with any treatment), missing CA data, missing survival data, or interfacility transport were excluded.

### Measures

Transport time was defined as the time from EMS leaving trauma scene to hospital arrival. Extrapolation exceeding 60 min was counted as 60 min. Patients with prehospital return of spontaneous circulation (ROSC) are usually not considered for termination of resuscitation. Patients without ROSC were separately analyzed. Primary outcome was set as survival to discharge.

### Data analysis

Continuous variables and ordinal variables were described using medians with interquartile ranges (IQR). Categorical variables were described with numbers and percentages. We conducted a binomial log-linear regression analysis to evaluate the association between transport time and survival. We first used cubic spline curves with four knots to draw the association of transport time with chances of survival for every minute and risk ratio (RR) for survival. We estimated 95% confidence intervals (CI) for both; when we estimated RRs, we used 15-min transport time as a reference. Then, we estimated the RR for survival dividing the transport time into a dichotomy (i.e., less than 15 min vs 15 min or greater) and used the category of less than 15 min as a reference. Statistical analysis was performed using STATA version 16 (StataCorp LP, College Station, TX).

## Results

### Study participants

**Fig. 1:**
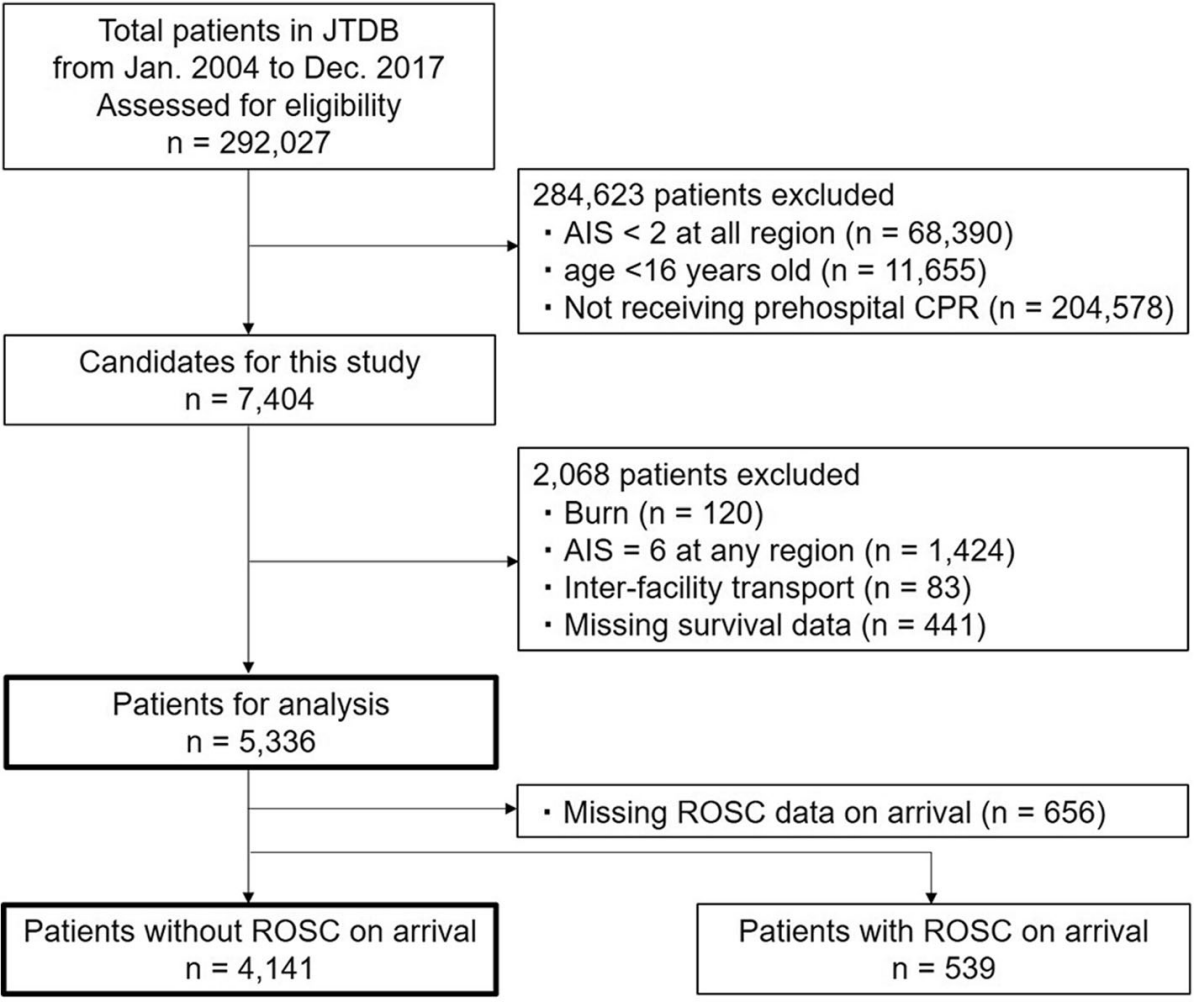
Flow diagram of patients analyzed. AIS: Abbreviated Injury Scale; CA: cardiac arrest; CPR: cardiopulmonary resuscitation; JTDB: Japan Trauma Data Bank; ROSC: return of spontaneous circulation

Of 292,027 patients registered in the JTDB during the 14-year study period, 7404 trauma patients met the inclusion criteria. After exclusion, the final analysis contained 5336 patients. Patients with sustained TCA (4141 patients) were analyzed separately from overall patients (including prehospital ROSC; 5336 patients) for survival and transport time association (Fig. 1). Table 1 shows the patient characteristics. The median age of all patients was 53 years (IQR 36–70), and 67.2% were male. Accident was the most common cause of trauma (53.3%), and blunt trauma was far more common than penetrating trauma (94.5 vs 5.5%). Their median ISS was 29 (IQR 22–41). Median transport time in overall patients was 11 min (IQR 6–17 min). For patients with sustained TCA, median transport time for survivors (8 min, IQR 4–13 min) was shorter than non-survivor (11 min, IQR 6–17 min). Median EMS encounter time (patient-provider contact at the scene to hospital arrival) in overall patients was 29 min (IQR 18–60 min). Overall median probability of survival (TRISS method estimation) was 2.0% (IQR 0.9–6.8%), and 4.5% survived until discharge. Patients’ characteristics were similar for the overall cohort and sustained TCA, except that sustained TCA had only a 1.2% rate of survival until discharge. Patients with ROSC (*n* = 539) at arrival had longer transport time than the overall cohort (13 min, IQR 8–24 min) but with 28.0% rate of survival until discharge.

**Table 1:** Characteristics of patients with traumatic cardiac arrest

	All (*n* = 5336)	Sustained cardiac arrest at arrival (*n* = 4141)	ROSC on arrival (*n* = 539)
Age, median years [IQR]	53 [36–70]	52 [36–69]	62 [42–73]
Sex male, n (%)	3588 (67.2%)	2780 (67.1%)	386 (71.6%)
Cause of trauma, n (%)			
Accident	2844 (53.3%)	2084 (50.3%)	361 (67.0%)
Industrial accident	300 (5.6%)	223 (5.4%)	40 (7.4%)
Self-injury	1661 (31.1%)	1417 (34.2%)	96 (17.8%)
Assault	101 (1.9%)	80 (1.9%)	10 (1.9%)
Other	430 (8.1%)	337 (8.1%)	32 (5.9%)
Blunt trauma	4961 (94.5%) ^a^	3847 (94.2%) ^b^	501 (94.9%) ^c^
Penetrating trauma	286 (5.5%)^a^	235 (5.8%)^b^	27 (5.1%)^c^
ISS, median [IQR]	29 [22–41]	29 [22–41]	26 [19–36]
Ps using TRISS method, median [IQR]	2.0% [0.9–6.8%]	1.7% [0.8–5.0%]	15.5% [4.8–45.8%]
EMS transport time, median minutes [IQR]	11 [6–17]	11 [6–17]	13 [8–24]
Survivor, median minutes [IQR]	11 [6–20]	8 [4–13]	13 [8–22]
Non-survivor, median minutes [IQR]	11 [6–17]	11 [6–17]	12 [8–24]
EMS encounter time, median minutes [IQR]	29 [18–60]	29 [18–60]	33 [20–60]
Survival at discharge, n (%)	239 (4.5%)	48 (1.2%)	151 (28.0%)

^a^ evaluated in 5247 patients. ^b^ evaluated in 4082 patients. ^c^ evaluated in 528 patients. *EMS* Emergency medical services, *IQR* Interquartile range, *ISS* Injury Severity Score, *Ps* Probability of survival, *ROSC* return of spontaneous circulation, *TRISS* Trauma Injury Severity Score. Missing ROSC data on arrival for 656 patients. EMS transport time was defined as the time from EMS leaving trauma scene to hospital arrival. EMS encounter time was defined as the time from patient-provider contact at the scene to hospital arrival

### Emergency medical services encounter time and survival

**Fig. 2:**
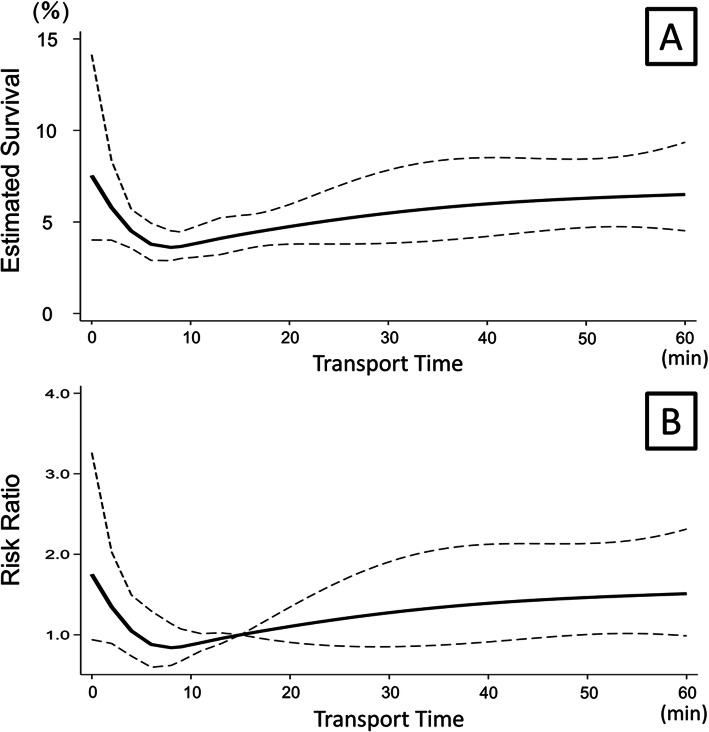
Association between emergency medical service transport time and survival in overall traumatic cardiac arrest patients (including return of spontaneous circulation at arrival) is drawn in the solid line with 95% confidence interval in dotted line. **A**. Estimated survival over transport time. **B**. Risk ratio over transport time with 15 min used as the reference time (RR =1.0). RR: risk ratio

When we examined the association between transport time and survival in all patients (including ROSC at arrival), estimated survival decreased from 8% at the beginning of transport time to 4% at 6–17 min, then remained steady at around 6% after 18 min. The RR decreased from 1.75 at 0 min and bottomed at 8 min (RR: 0.84) then gradually recovered until around 40 min and plateaued (RR: 1.51 at 60 min). (Fig. 2).

**Fig. 3:**
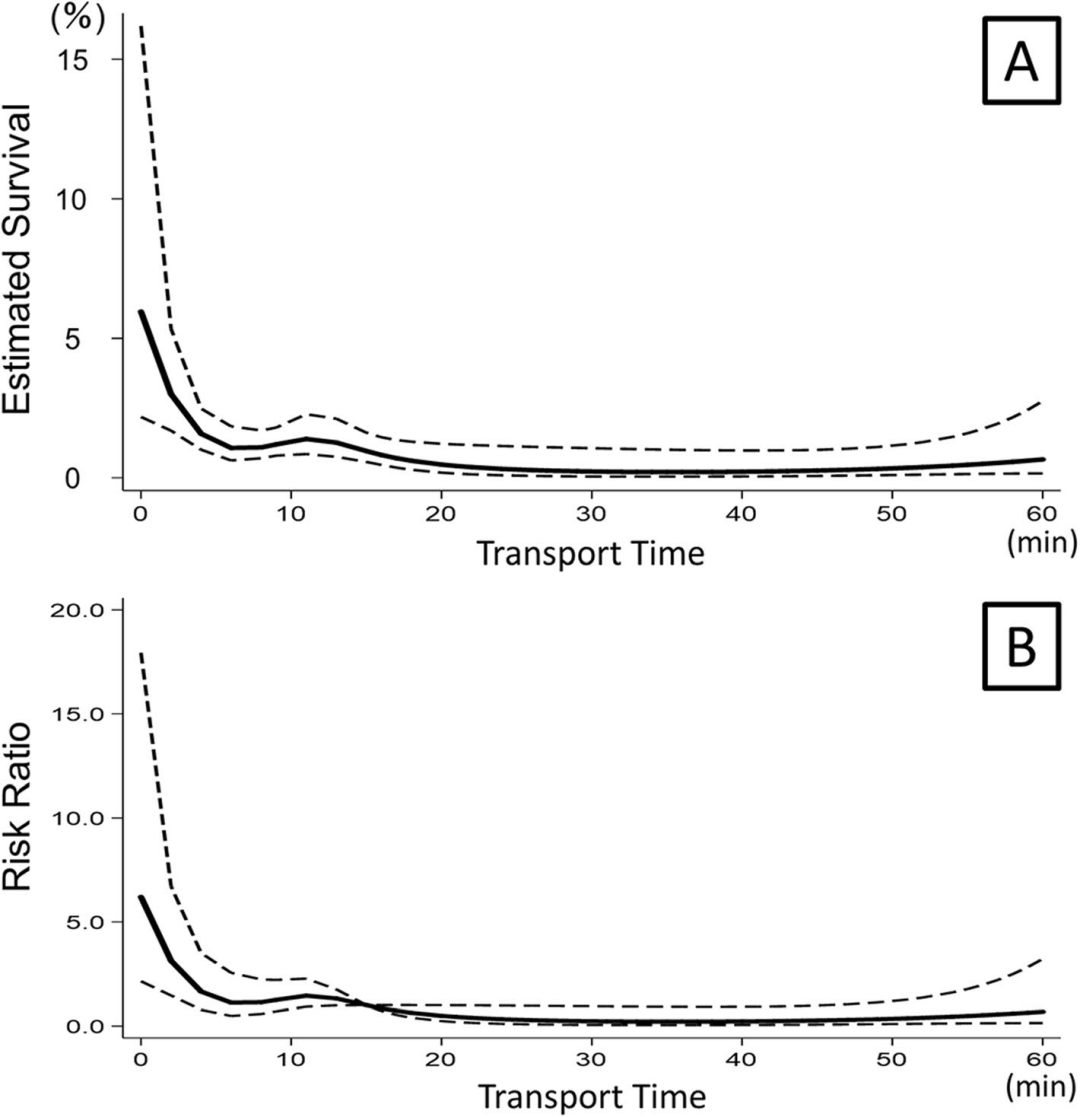
Association between emergency medical service transport time and survival in patients with sustained traumatic cardiac arrest is drawn in the solid line with 95% confidence interval in dotted line. **A**. Estimated survival over transport time. **B**. Risk ratio over transport time with 15 min used as the reference time (RR =1.0). RR: risk ratio

When this association was examined in patients who remained in TCA status at arrival, estimated survival decreased from 6% at the beginning of transport time (0 min) to 1% after 15 min of transport time. The RR decreased from 6.18 at 0 min to 1.0 (reference point) at 15 min and below 1.0 immediately after 15 min. After 15 min of transport time, the curves almost flattened and both estimated survival (0–1%) and RR remained low until 60 min. (Fig. 3).

When we divided the transport time into a dichotomy, the RRs for survival for transport time ≥ 15 min compared with transport time < 15 min were 1.29 (1.00–1.66) for all patients including ROSC at arrival, 0.47 (0.23–0.97) for the patients who remained in sustained TCA at arrival, and 1.01 (0.77–1.32) for the patients with ROSC.

## Discussion

In this study using a large multi-institutional database, we demonstrated an association between EMS transport time and survival after TCA. For patients with sustained TCA at arrival, the estimated chances of survival declined rapidly with time. After 15 min of transport time, the estimated survival rate of sustained TCA patients flattened and remained below 1%. Time can be one reasonable factor for considering termination of resuscitation in patients with sustained TCA, although clinical signs of life and type and severity of trauma should be taken into account clinically. To our knowledge, ours is the first report to define the survival curve of patients with TCA in a large, multi-institutional cohort.

The mortality of patients with TCA can vary depending on the treatment options available. Some TCA patients in our study experienced ROSC early in their contact with EMS or during transport, affecting the overall patient survival curve even beyond 15 min of transport time, with estimated survival estimates remaining at 5 to 6%. The basic life support and advanced procedures paramedics are allowed to use in the system [6], such as advanced airway management, intravenous line establishment, and administration of adrenaline, may contribute to some patients’ survival. TCA patients who had these effective procedures performed at the scene or during the transport with prehospital ROSC had a much higher proportion of survival at discharge compared to sustained TCA. Prehospital ROSC patients may have had airway/respiratory/neurological deterioration rather than exsanguination, conditions which can be stabilized with on-scene airway and respiratory management. However, we could not explore which type of injury caused TCA due to the limits of the database. Determining which of several early treatment options are the most effective for increasing survival chances beyond the 15 min time window may depend on the type of trauma endured, and will require further study. In the case of physician-staffed EMS, physicians can perform additional prehospital procedures, such as chest tube insertion and administration of red blood cells or blood plasma, that are potentially beneficial for patients with TCA [7–9]. Although still controversial, thoracotomy in the ED for open-chest CPR may increase the chances of survival, as demonstrated when Endo and colleagues compared open-chest versus closed-chest CPR in TCA patients with signs of life upon hospital arrival [10]. Resuscitative endovascular balloon occlusion of the aorta is increasingly used in patients with severe trauma and may also contribute to successfully treating TCA [11]. Further study is required to test whether these procedures and strategies may increase the critical time limit for TCA.

Despite advances in trauma care, survival rates for TCA patients who arrive at a trauma center with no signs of life remain low [12]. A 2012 systematic review demonstrated a 3.3% mortality rate for adult patients with TCA [13]. Duration of CPR is one crucial concern for terminating resuscitation, however the mechanism of injury and the physiological status of the patient should also factor into the decision to terminate resuscitation after TCA. As shown in our study, ROSC before arrival at the ED is a predictor for survival, and longer transport times did not reduce survival-to-discharge when ROSC was observed, indicating continued efforts should be devoted to these cases. Other studies have shown that an initial shockable rhythm, agonal breathing, or a corneal/pupil reflex may indicate improved survival odds [14–17]. Pulseless electrical activity (PEA) or preserved cardiac wall motion observed by ultrasound examination may also be predictive of survival and can be easily performed in the ED [18]. Different types of injury can cause TCA, and patients with obstructive causes (such as tension pneumothorax) tend to have a higher chance of survival compared with patients whose TCA was caused by exsanguination or severe brain injury [7, 19]. However, consideration of the type of injury was outside the scope of this study.

Making the decision to terminate resuscitation can be difficult. Although few TCA patients survive, even after more than 15 min of CPR [20], the number of patients who could be salvaged may be underestimated. However, showing the survival curve is necessary for the effective use of limited medical resources. In our emergency medical service system, paramedics are not legally permitted to terminate resuscitation in the field. The decision to terminate resuscitation is prolonged to reach the hospital, since some procedures and assessments are only available in the ED. Physicians should thoroughly examine clinical signs predictive of survival prior to considering resuscitation time when deciding to terminate sustained TCA.

The current study has some limitations. We could not obtain the exact duration of CA, and instead used EMS transport time as a surrogate, for most of the patients with sustained TCA actual CA duration is longer than this time window. However, this study demonstrated a survival curve which may be used as one factor to determine termination of resuscitative efforts after TCA. Our emergency service system generally does not allow cessation of resuscitation on scene. Therefore, the survival curve is mainly targeted for emergency department physicians or surgeons when considering termination of resuscitation. Transport times were fairly short, presumably because the majority of participating institutions are located in urban areas, conditions which may not be practical in rural areas. However, the initial time course may still be relevant for all TCA patients. Further study is needed to examine the effectiveness of prolonged efforts for patients in rural areas. Additionally, we could not obtain data for some potential indicators of survival such as pupillary reflexes or spontaneous movement. These indicators should be considered before withholding or withdrawing CPR. We also did not consider outcomes after advanced resuscitative treatments such as open-chest CPR or resuscitative endovascular balloon occlusion of the aorta. Neurological outcomes were not considered in our study, and some survivors of TCA have neurological impairment. Finally, as with all retrospective studies, data integrity, validity, and ascertainment bias are potential limitations.

## Conclusions

In this large database study, we observed that the chances of survival for sustained TCA declined rapidly while the patient is transported with CPR support. There was less than 1% survival for patients receiving CPR after sustained TCA with transport time > 15 min. Time should be one reasonable factor for considering termination of resuscitation in patients with sustained TCA, although clinical signs of life and type and severity of trauma should be taken into account clinically.

## Data Availability

The data for the study is obtained from Japan Trauma Data Bank (https://www.jtcr-jatec.org/traumabank/index.htm); the authors do not have permission to share data.

## Abbreviations

ACSCOT: American College of Surgeons Committee on Trauma
AIS: Abbreviated Injury Scale score
CA: Cardiac arrest
CI: Confidence interval
CPR: Cardiopulmonary resuscitation
ED: Emergency department
ELST: Emergency life-saving technician
EMS: Emergency medical services
IQR: Interquartile range
ISS: Injury Severity Score
JTDB: Japan Trauma Data Bank
NAEMSP: National Association of EMS Physicians
PEA: Pulseless electrical activity
ROSC: Return of spontaneous circulation
RR: Risk ratio
TCA: Traumatic cardiac arrest
TRISS: Trauma Injury Severity Score
